# Inhibition potential of margolonone and isomargolonone against the dengue virus protease using molecular modeling approaches

**DOI:** 10.3389/fbinf.2025.1517115

**Published:** 2025-03-26

**Authors:** Gourav Choudhir, Faiza Iram, Israil, Mohammad Shahid, Anas Shamsi, Md Imtaiyaz Hassan, Asimul Islam

**Affiliations:** ^1^ Centre for Interdisciplinary Research in Basic Sciences, Jamia Millia Islamia, New Delhi, India; ^2^ Department of Basic Medical Sciences, College of Medicine, Prince Sattam Bin Abdulaziz University, Al-Kharj, Saudi Arabia; ^3^ Center for Medical and Bio-Allied Health Sciences Research, Ajman University, Ajman, United Arab Emirates

**Keywords:** NS3 protease, integrating modeling, natural product, binding energy, nontoxic

## Abstract

**Background:**

Dengue is a mosquito-borne viral disease with no cure. Inhibiting key enzymes vital in replication could manage the dengue virus infection. This study investigated the potential of margolonone and isomargolonone from *Azadirachta indica* to inhibit dengue virus replication.

**Methods:**

The 3D structure of margolonone and isomargolonone were obtained from the PubChem database. The drug-likeness properties of these molecules were performed using a Swiss-ADME server. The molecular docking and molecular dynamics simulation assessed binding affinity and interactions.

**Results:**

The drug-likeness of parameters showed that Margolonone and isoMargolonone showed zero violation of Lipinski rules. Docking simulations showed that both compounds bind to the active site of a critical enzyme (NS3 protease) essential for viral replication. Molecular dynamics simulations suggested that isomargolonone may bind more stably to NS3 than margolonone. Additionally, MMPBSA analysis showed that Margolonone does not show favorable binding energy.

**Conclusion:**

These findings warrant further investigation of isomargolonone as a potential anti-dengue drug. Further *in-vitro* and *in-vivo* evaluations need to be done before accepting it as drug molecules.

## 1 Introduction

Dengue is a viral disease that is transmitted by Aedes mosquitoes. The dengue virus belongs to Flaviviridae, which causes severe life-threatening shock and hemorrhagic fever in humans. In India and other parts of the world, dengue has claimed lives over the years, and people have suffered in every state of India (https://ncvbdc.mohfw.gov.in/) (Hasan et al., 2016). According to the Centers for Disease Control and Prevention (CDC), more than 400 million people are affected by the dengue virus, which can increase yearly (https://www.cdc.gov/). Around 40,000 people die due to dengue infections (https://www.cdc.gov/). Finding a cure for the dengue virus is urgently needed to save the lives of millions of people around the world.

Dengue virus is a positive single-stranded RNA virus. The survival and replication of the dengue virus depend on the cheerful cooperation of structural and nonstructural proteins (Sinha et al., 2024). Structural protein assists in the formation of viral capsid and host membrane attachment. Nonstructural proteins participate in the dengue virus replication (Sinha et al., 2024). Nonstructural proteins are named NS1, NS2A, NS2B, NS3, NS4, and NS5 (Sinha et al., 2024). These are all nonstructural proteins that participate in the successful replication of the dengue virus. The dengue virus matures in the host body by processing RNA-encoded polypeptide protease, defined as NS2B/NS3 protease (Lin et al., 2017). NS2B/NS3 protease plays a part in the pathogenesis of dengue virus infection via RNA replication in the host cell (Diamond and Pierson, 2015). NS2B/NS3 is essential for virus replication and assembly. NS3 acts as a dengue virus protease, whereas NS2B is a cofactor stabilizing the NS3 (Erbel et al., 2006; Norshidah et al., 2023). The NS3pro domains in both structures share a fold similar to chymotrypsin. This fold comprises two beta-barrel structures, each formed by six beta strands. The catalytic triad, composed of histidine at position 51, aspartic acid at position 75, and serine at position 135, is situated at the cleft where the two barrels meet (Erbel et al., 2006).

Natural products have been used to remedy different diseases for a long back. Metabolites are rich in structural and functional diversity and play vital roles in mitigating infections of various groups of viruses. Natural products interfere with multiple groups of viruses via their replication, host attachment, and host body maturation (Elsebai et al., 2016; Gao et al., 2019). (Kitazato Kaio, 2007; Elsebai et al., 2016; Gao et al., 2019). Plants have antiviral potential against different virus groups (Mukhtar et al., 2008). Inhibition of NS2B/NS3 protease could be beneficial for managing dengue infections. In the current study, we have explored the binding affinity and stability of dengue NS3 protease and the structural analog of two compounds named margolonone and isomargolonone using combined molecular docking, molecular dynamics simulation, and MMPBSA approaches to assess stability and binding affinity.

## 2 Materials and methods

### 2.1 Druglikeness and toxicity analysis of selected bioactive compounds

The drug-likeness of selected compounds has been assessed to determine whether the drug is safe to consume and has few/no side effects. Several parameters, such as absorption distribution, metabolism, and excretion, have been considered to assess the effectiveness of drugs. Drugs should have optimum values of ADME parameters to provide better treatment with fewer side effects. The SWISS-ADME server performed the ADME analysis (Daina et al., 2017). Further toxicity of these compounds was predicted using the ProTox 3.0 server, which includes organ toxicity, target organs, toxicity endpoints, and relevant molecular pathways involved (Banerjee et al., 2024).

### 2.2 Molecular docking

Molecular docking is a computationally based technique that predicts the affinity and interactions of a drug with macromolecules. Molecular docking assists in identifying the affinity of drug targets and molecules based on the properties of interacting molecules and macromolecules (Agu et al., 2023).

#### 2.2.1 Receptor/protein preparation

The 3D coordinates of NS2B/NS3 [PDB ID: 2FOM] were retrieved from the RCSB database (https://www.rcsb.org/). NS2B/NS3 was processed by removing the NS2B and water molecules. Additionally, the clean geometry module of Discovery Studio was applied to optimize the structure (San Diego, CA, United States) and saved into pdb format. The optimized pdb was processed in MGL tool 1.5.6 (Scripps, California). The hydrogen atom and Kollaman charged were added and saved in the pdbqt file. The pdbqt file was used for docking studies.

#### 2.2.2 Ligand preparation

The 3D structure of Margolonone (CID = 189,726) and isomargolonone (CID = 189,727) was retrieved in SDF format from the PubChem database (Kim et al., 2023). Further, margolonone and isomargolonone were processed in open babble build in PyRx by considering the universal force field and steepest descent algorithm (Dallakyan and Olson, 2015). The optimized files are saved in pdbqt format.

#### 2.2.3 Ligand and receptor/protein docking

The molecular docking was performed at the atomic map at 1 Å using AutoDock Vina (Trott and Olson, 2010). The receptor of the grid box was set on the catalytic triad of the active site, which is essential for catalytic activity. The grid box dimensions were set as 16 Å × 16 Å × 16 Å. The center point grid box was set as X = −2.178, Y = −11.059 and Z = 15.625. The extensiveness value was set to 16. The binding energy of margolonone and isomargolonone was analyzed, and further interaction was visualized using the receptor-ligand interactions module (San Diego, CA, United States).

### 2.3 Molecular dynamics simulation

Molecular dynamics (MD) simulations were performed to model the atomic-level behavior of The stability of margolonone-NS3 and isomargolonone-NS3 complexes over time. The simulations used GROMACS 2018.7 with the AMBER99SB-ILDN force field to represent interatomic interactions (Berendsen et al., 1995). The ACPYPE server-generated ligand topology with GAFF2 force field for selected molecules (Sousa Da Silva and Vranken, 2012). Each system was solvated in a cubic box with water molecules neutralized with 150 mM NaCl to simulate physiological salt concentrations. Energy minimization was performed using the steepest descent algorithm. Electrostatic interactions were calculated using the Particle Mesh Ewald (PME) method, while short-range van der Waals and Coulombic interactions were calculated directly with a 9 Å cutoff. The systems were equilibrated in two stages: a 100 ps NVT simulation for solvent and ion relaxation and a 10 ns NPT simulation with gradual release of restraints on complexes to allow for conformational adjustment. Temperature and pressure were maintained at 310.15 K and 1 atm, respectively, using a Berendsen thermostat and a Parrinello-Rahman barostat. Production MD simulations of 100 ns were conducted using the LeapFrog integrator with a 2 fs time step and periodic boundary conditions. Trajectory analysis was performed using GROMACS tools and custom Python scripts (Choudhir et al., 2025). The stability of margolonone-NS3 and isomargolonone-NS3 complexes was assessed using various parameters such as RMSD, RMSF, Rg, SASA, hydrogen bond, and free energy landscapes. The results of complexes were compared with the control (without Ligand or apo form) of NS3. The MDS was performed for the 100 ns to assess the interaction and stability of the molecules. The free energy landscape was also performed according to the protocol discussed in the previous study (Choudhir et al., 2025).

### 2.4 MMPBSA analysis

The MMPBSA method is a popular computational tool for estimating the binding affinity between protein-ligand interactions (Kumari et al., 2014). The MMPBSA-based binding energy depends on polar and nonpolar entities involved in interactions. The last 10 ns trajectories were used for MMPBSA analysis (Choudhir et al., 2025).

## 3 Results

### 3.1 Druglikeness and toxicity of selected bioactive compounds

**FIGURE 1:**
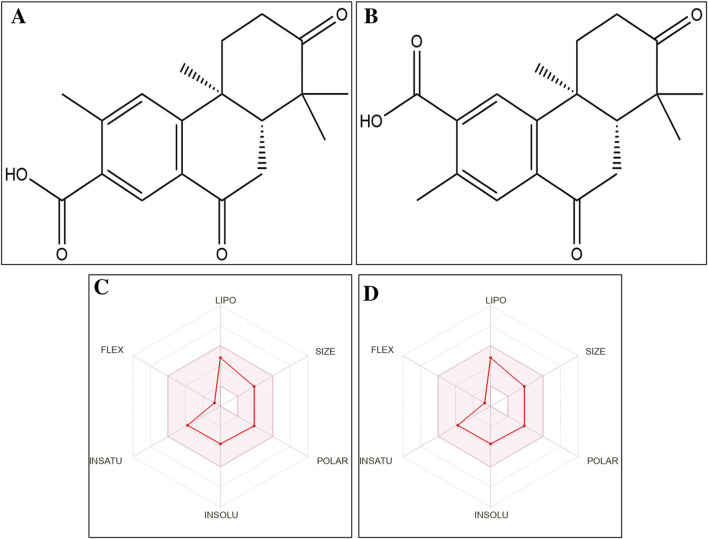
**(A)** 2D structure of margolonone **(B)** 2D structure of isomargolonone **(C)** Bio radar diagram of margolonone **(D)** Bio radar diagram of isomargolonone.

Drug likeness and toxicity are essential for evaluating the bioactive compounds of potential drugs with desired pharmacokinetics and pharmacodynamic properties and their side effects. Drug likeness parameters, including size and solubility, are essential for drug actions. The margolonone and isomargolonone have zero violation of Lipinski’s rule of five. Margolonone and isomargolonone have a molecular weight of 314.38 g/mol and are structurally different. Most parameters showed similar values and had one group’s position differences (Figure 1; Supplementary Table 1). The LD50 value of margolonone and isomargolonone was 570 mg/kg. Additionally, margolonone and isomargolonone do not possess organ toxicity as well as inactive towards cytotoxicity, mutagenesis, and immunotoxicity. The different toxicity parameters are given in the Supplementary Table 2. Both molecules share a phenanthrene core with tetramethyl and dioxo substitutions, differing only in the position of the carboxylic acid group. Margolonone has the carboxyl at position 2, while isomargolonone has it at position 3, leading to regioisomeric differences affecting polarity and possible biological interactions.

### 3.2 Molecular docking

**FIGURE 2:**
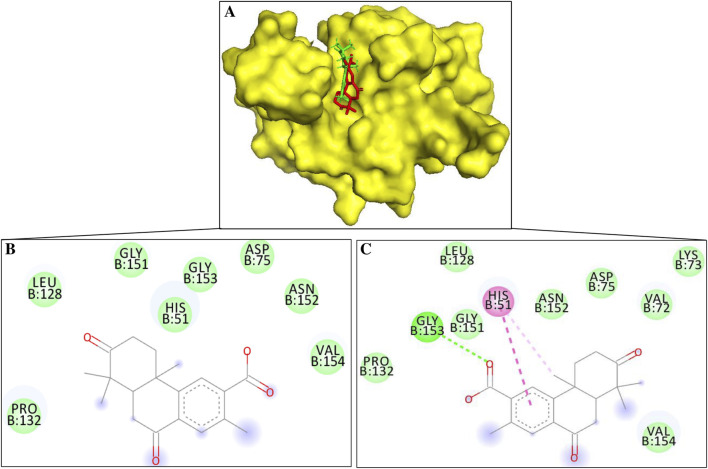
**(A)** Surface diagram of NS3-margolonone (Red color) – isomargolonone (Green color) **(B)** 2D interaction of NS3-margolonone **(C)** 2D interactions of NS3-isomargolonone.

NS3 is a nonstructural protein essential for the dengue virus replication. The inhibition of NS3 interactions with the structural and nonstructural protein can reduce the stability during the initiation and maintain homeostasis during the replication. A potential drug could interact with the amino acid and participate in catalytic processes of NS3, such as His51, Asp75, and Ser135 (Erbel et al., 2006). The interaction of the inhibitor with the catalytic triad reduces/diminishes the activity of NS3 protease (Erbel et al., 2006). The binding affinity and interaction analysis showed that margolonone and isomargolonone have a descent binding affinity with the NS3 and also formed interactions with the essential amino acid residues involved in the function of NS3. The margolonone and isomargolonone adopt slightly different binding conformations while binding with the NS3, possibly due to their structural differences (Figure 2A).

Margolonone showed a binding energy of −6.8 kcal/mol. NS3 and Margolonone interact via the van der Waals contacts. The amino acid residues include His51, Asp75, Leu128, Pro132, Gly151, Asn152, Gly153, and Val154 (Figure 2B). Among the catalytic triad, margolonone interacts with His51 and Asp75.

Isomargolonone has a binding energy of −7.0 kcal/mol. The isomargolonone formed diverse interactions with NS3, including Pi-Pi stacking, hydrogen bonds, and Van der Waals interactions. Amino acid residues of NS3 such as Val72, Lys73, Asp75, Leu128, Pro132, Gly132, Gly151, Asn152, and Val154 via van der Waals contact. His51 interacts via the pi-alkyl and pi-pi stacking interaction, and one hydrogen bond is formed between the isomargolonone and Gly153 (Figure 2C). The interactions of bioactive compounds with the active site of NS3 could diminish the biological functions.

Margolonone and isomargolonone interact with amino acid residues in the catalytic triad. The selected molecules interact with the His51 using hydrogen bond and pi interactions. The differences in the binding conformations lead to differences in the binding energy. Involvements of interactions with the various amino acid residues may reduce/diminish NS3 catalytic activity. The stability and interactions of NS3 and docked compounds were also assessed using molecular dynamic simulation.

### 3.3 Molecular dynamics simulation

Molecular Dynamics Simulation (MD) is a powerful technique that can measure the stability and interactions of interacting entities under physiological conditions. MD Simulation elucidates the structural and functional parameters of interacting entities under the selected parameters. Stability and interactions analysis of the NS3-margolonone and NS3-isomargolonone elucidates using RMSD, RMSF, Rg, SASA, hydrogen bond, and free energy landscape. The above-selected MD Simulation parameters provide details of the dynamics and stability of NS3 in compounds bound state and apo form.

#### 3.3.1 Root mean square deviation (RMSD) analysis

**FIGURE 3:**
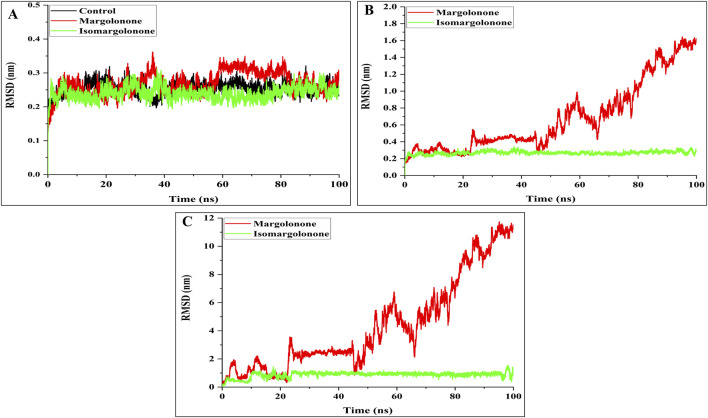
Stability analysis of NS3 during the simulation time **(A)** Backbone RMSD **(B)** complex RMSD **(C)** ligand RMSD.

RMSD is essential for assessing protein stability and dynamics in simulating macromolecules’ bound and apo states. The binding of ligands with the proteins impacts the macromolecule’s structural and functional properties. Backbone RMSD analysis showed that the backbone RMSD of apo form and bound state with margolonone and isomargolonone were found in the 0.2–0.3 nm (Figure 3A). The RMSD of control and isomargolonone does not fluctuate much throughout the simulation, whereas in the case of the margolonone backbone, RMSD showed slight fluctuations around 60–80 ns. Further complex and Ligand RMSD analysis showed that isomargolonone has a tight binding compared to margolonone (Figures 3B, C). Due to loose binding, the margolonone emerges from the NS3 binding after the 40 ns simulation. The RMSD of backbone, complex, and ligand analysis showed that isomargolonone has a more stable binding than margolonone.

#### 3.3.2 Radius mean square fluctuation (RMSF) analysis

**FIGURE 4:**
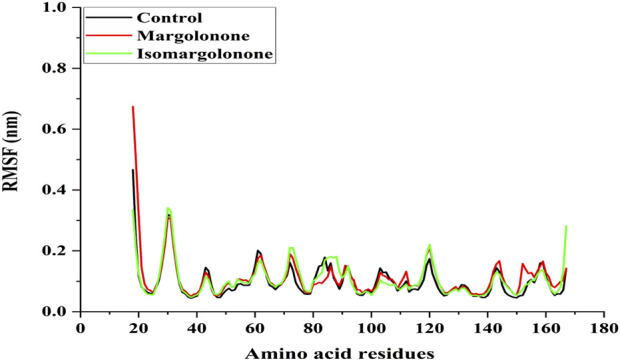
RMSF analysis of NS3 in apo form and bound state with margolonone and isomargolonone.

RMSF measures the fluctuation of amino acid residues present in the proteins. Amino acid residues offer a space to accommodate interacting ligands by changing their conformation. RMSF analysis showed that the binding of margolonone and isomargolonone does not significantly impact RMSF intensity. The maximum RMSF values were found below 0.3 nm expected N-terminal in control and bound form with margolonone and isomargolonone (Figure 4).

#### 3.3.3 Radius of gyration (Rg) analysis

**FIGURE 5:**
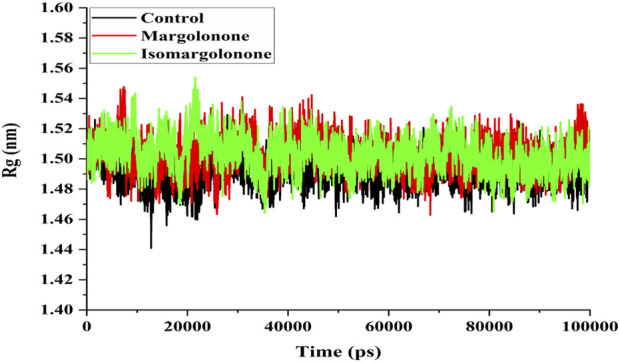
Rg analysis of NS3 in the apo form and bound state with margolonone and Isomargolonone.

The Rg measure of the compactness of protein can be further elaborated on how tightly the protein is packed. The compactness of proteins is essential for regulating their structure and functional properties (Subramanian Manimekalai et al., 2016). Rg analysis showed that the Rg values of NS3 in apo form and bound form lie between 1.48 and 1.54 nm. The Rg values of the apo and ligand-bound states of NS3 do not significantly differ. However, the Rg values in the control were slightly lower throughout the simulation (Figure 5).

#### 3.3.4 Solvent accessible surface area (SASA) analysis

**FIGURE 6:**
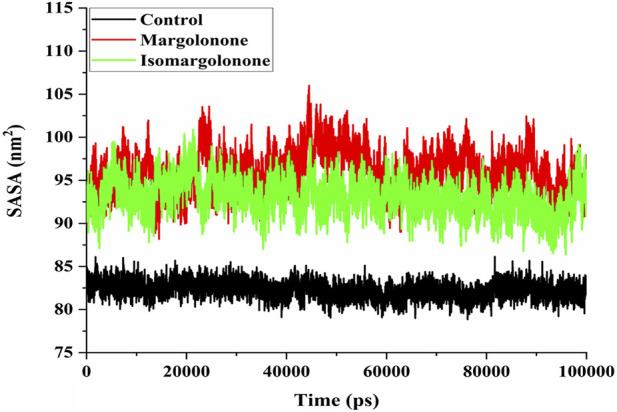
SASA analysis of NS3 in the apo form and bound state with margolonone and Isomargolonone.

SASA measure is a measure of the surface area of a protein structure that is accessible to solvent molecules in biological systems. SASA provides valuable conformation insights into protein and how it changes over time concerning simulation. SASA analysis showed that 80–85 nm^2^ had steady values in the control case. In the case of margolonone and isomargolonone, SASA values showed higher values (90–100 nm^2^) than those of the control (Figure 6). Margolonone showed higher SASA values than isomargolonone.

#### 3.3.5 Hydrogen bond analysis

**FIGURE 7:**
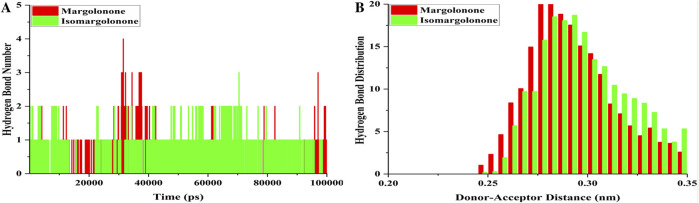
Hydrogen bond analysis **(A)** Hydrogen bond formed **(B)** hydrogen bond distribution of NS3 bound with margolonone and Isomargolonone.

Hydrogen bonds participate in drug binding and specificity during drug interactions with targets (Gancia et al., 2001). The hydrogen bond number analysis showed that hydrogen bonds formed throughout the simulation (Figure 7A). At least one hydrogen was maintained most of the time during the simulation. Four hydrogen bonds formed in the case of margolonone, and the case of isomargolonone formed 3 hydrogen bonds maximum in the simulation time. During the simulation, 2–3 hydrogen bonds formed at some point (Figure 7A). The hydrogen bond analysis showed that hydrogen bonds were distributed in the donor-acceptor distance of 0.25–0.35 nm. The maximum number of hydrogens distributed is 0.27–0.30 nm within the donor-acceptor distance. The maximum hydrogen bond is distributed around 20 bonds (Figure 7B).

#### 3.3.6 Eigenvector and free energy landscape analysis

**FIGURE 8:**
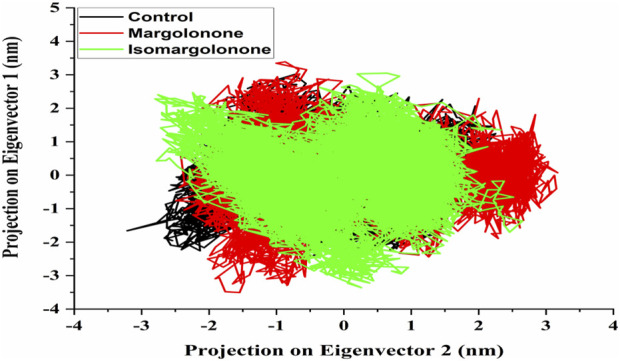
Eigenvector of c-alpha carbon of NS3 in apo form and bound state with margolonone and Isomargolonone.

**FIGURE 9:**
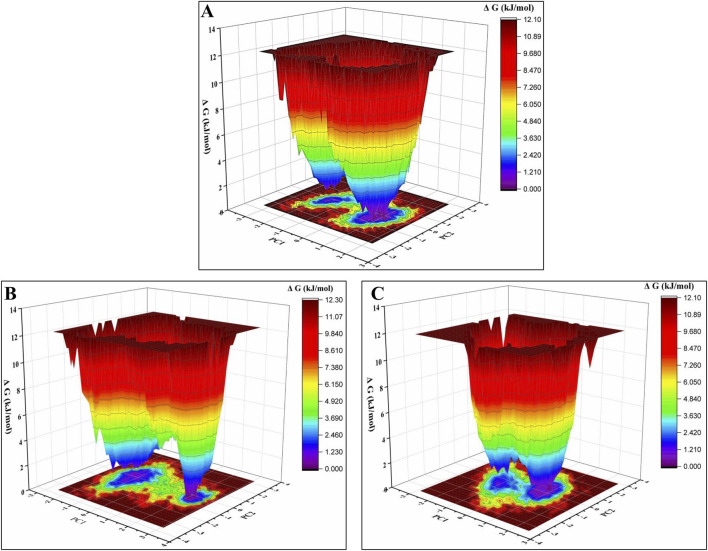
Free energy analysis of **(A)** Apo form of NS3 **(B)** NS3- margolonone **(C)** NS3- isomargolonone.

An essential dynamics analysis is used to identify the critical motions differentiating between the control, margolonone, and isomargolonone complexes. This analysis provides the eigenvectors representing the dynamical differences, and further covariance matrix examination was performed to decipher the essential dynamics. The eigenvector analysis showed that eigenvectors overlap most of the time. The binding of Margolonone and isoMargolonone showed a slight difference in eigenvectors compared to the control (Figure 8). Further FEL analysis showed free energy lies in the 12.10–12.30 kJ/mol range. The Margolonone showed free energy was 12.30, whereas the free energy of control and isomargolonone was found to be 12.10 kJ/mol. In all three cases, two energy minima were found: one was deep, and the other one shallow (Figure 9).

### 3.4 MMPBSA analysis

GROMACS g_mmpbsa was used to estimate the binding free energy of NS3-margolonone and NS3-isomargolonone complexes. The trajectories of MDS showed that NS3 and Margolonone showed a positive binding, as the complex and Ligand RMSD analysis indicated. The Margolonone had little affinity with NS3, which was expelled at 40 ns. In the case of NS3 and isomargolonone complex, the binding energy in negative values means they showed a higher affinity to each other. MMPBSA results are shown in Table 1.

**TABLE 1:** Binding free energy components for Margolonone-NS3 and Isomargolonone-NS3 complexes using MMPBSA.

Energy (kJ/mol)	Margolonone	Isomargolonone
Van der Waal Energy	0.000 ± 0.000	−86.719 ± 21.502
Electrostatic Energy	−0.011 ± 0.298	−5.715 ± 7.757
Polar Solvation Energy	22.098 ± 23.345	46.470 ± 15.161
SASA Energy	−0.267 ± 1.124	−9.531 ± 1.695
Binding Energy	21.820 ± 23.127	−55.494 ± 12.579

## 4 Discussion

Drug development is a lengthy process in which many molecules must be screened for various parameters to get a potential molecule with desired drug properties and side effects. The alliance between the experimental and computational approaches can improve drug discovery and reduce drug development costs (Szymański et al., 2011). Integrating results from drug-likeness, molecular docking, molecular dynamic simulation, and MMPBSA showed that the chemical spaces in the molecules play vital roles in the binding of drugs (Carlsson and Luttens, 2024). However, molecular docking results showed that margolonone has a slightly better binding affinity than isomargolonone. Molecular dynamics simulation and MMPBSA analysis showed that isomargolonone formed a stable complex and favorable binding energy compared to margolonone. Margolonone and isomargolonone belong to the diterpenoids class of plant secondary metabolites (Ara et al., 1989). Previous studies showed that margolonone had decent binding energy with the antibacterial and antifungal targets using drug-likeness, molecular docking, and MD simulation (Abdalla et al., 2021; James et al., 2024). Selected compounds have been reported to have diverse biological activities such as antibacterial, antiviral, anticancer, antioxidant, and so on (Ara et al., 1989; Uzzaman, 2020). These compounds are found in the *Azadirachta indica,* which was reported as an antiinfection agent and manages diabetes, cancer, and obesity (Uzzaman, 2020). Isomargolonone showed a higher affinity and interactions with the key amino acids of acetylcholinesterase enzymes with Alzheimer’s disease (Choudhir et al., 2022). Isomargolonone also showed anti-schistosomiasis activity via the formation of stable interactions and stability with the thioredoxin glutathione reductase (TGR) enzyme (Onile et al., 2024).

Margolonone and isomargolonone share the same core structure of a tetrahydrophenanthrene with four methyl groups and two ketone groups. The key difference lies in the position of the carboxylic acid group, which is attached at the 2-position in margolonone, and the 3-position in isomargolonone provide significant differences in affinity and stability were found to be different in MDS and MMPBSA results. The difference in the stability and binding energy is due to differences in chemical space. The distribution of the functional group of structurally related molecules is correlated to an alternation in biological activities due to perturbation in physiochemical fate, which plays a vital role in biological activities (Lewandowski et al., 2020). The correlation of chemical structure and biological activity can be decoded using SAR (Fliri et al., 2005).

The structurally related anthocyanin with the changes in the position of the functional group showed a difference in the biological activities and color (Koss-Mikołajczyk et al., 2019). Chemical substructures sometimes have significant differences in biological activities (Klekota and Roth, 2008). The substructures have intense biological activity, are defined as privileged structures, and have less biological activity, which is defined as underprevalage (Klekota and Roth, 2008). Integrating the molecular modeling approach, such as molecular docking and MD simulation, can improve the selection of biologically active molecules among structurally similar molecules based on the interactions and stability. Our results provide insight into how structurally similar bioactive compounds with differences in the functional group can be distinguished in binding affinity and orientation. Applying molecular modeling can reduce the cost of selecting biological privilege substructures. Additionally, the computational design of substructure molecules can be a biological activity privilege compared to parent molecules or molecules present in nature.

## 5 Conclusion

The integration of results from different experiments of this study showed that isomargolonone has the potential to inhibit the NS3 protease via the formation of stable interactions. However, margolonone and isomargolonone have related structures and possible bioactivity differences due to the position of the small group, leading to differences in affinity with NS3. However, additional *in vitro* and *in vivo* investigations are necessary to assess the acceptance of isomargolonone as the clinical inhibitor of NS3 to manage dengue infections and its toxicity.

## Data Availability

The original contributions presented in the study are included in the article/Supplementary Material, further inquiries can be directed to the corresponding authors.
